# Risk factors for early recurrence after inguinal hernia repair

**DOI:** 10.1186/1471-2482-9-18

**Published:** 2009-12-09

**Authors:** Petra Lynen Jansen, Uwe Klinge, Marc Jansen, Karsten Junge

**Affiliations:** 1 Department of Surgery, University Hospital, RWTH Aachen, Germany; 2 Applied Medical Engineering, Helmholtz Institute, RWTH Aachen, Germany

## Abstract

**Background:**

Family history, male gender and age are significant risk factors for inguinal hernia disease. Family history provides evidence for a genetic trait and could explain early recurrence after inguinal hernia repair despite technical advance at least in a subgroup of patients. This study evaluates if age and family history can be identified as risk factors for early recurrence after primary hernia repair.

**Methods:**

We performed an observational cohort study for 75 patients having at least two recurrent hernias. The impact of age, gender and family history on the onset of primary hernias, age at first recurrence and recurrence rates was investigated.

**Results:**

44% (33/75) of recurrent hernia patients had a family history and primary as well as recurrent hernias occurred significantly earlier in this group (p = 0.04). The older the patients were at onset the earlier they got a recurrent hernia. Smoking could be identified as on additional risk factor for early onset of hernia disease but not for hernia recurrence.

**Conclusion:**

Our data reveal an increased incidence of family history for recurrent hernia patients when compared with primary hernia patients. Patients with a family history have their primary hernias as well as their recurrence at younger age then patients without a family history. Though recurrent hernia has to be regarded as a disease caused by multiple factors, a family history may be considered as a criterion to identify the risk for recurrence before the primary operation.

## Background

After primary hernia repair up to 10% recurrences are reported[1]. Mesh technique and the surgeons' experience are important factors to obtain good results. Nevertheless, the risk for hernia recurrence increases from repair to repair[2]. The question is if patients with more than one recurrence are not treated sufficiently or if their recurrences are based on disturbances of the extracellularmatrix.

Family history is an important predictors for the development of inguinal hernias[3] as well as for hernia recurrence[4] and indicates that genetic factors play a role for disease manifestation at least in a subgroup of hernia patients. Especially collagen genes are suspicious genes because studies on the biology of hernia formation suggest that disturbances in collagen metabolism contribute to high recurrence rates[5,6]. A decreased ratio of type I/III collagen is reported for inguinal, incisional and especially for recurrent hernias [7-10]. The collagen composition is of great importance not only for tissue integrity and resistance to tensile stress but also for sufficient tissue remodelling after tissue injury and disturbances of collagen expression could explain high recurrence rates [11-13]. Recently, type III collagene gene (Col3A1) polymorphisms were identified for patients with gastroesophageal reflux disease and hiatal hernias[14]. Northern blot as well as real time PCR analyses have shown changes in the expression of collagen genes [15-18].

Disturbed collagen expression can not only be caused by genetic alterations but also by exogenous factors, e.g. smoking and ascorbic acid deficiency. Moreover, it might be anticipated from diseases with gradual or late onset in life, e. g. atherosclerosis or hypertension, that the coincidence of multiple risk factors determines an individual's susceptibility. For the onset of inguinal hernia disease, smoking, comorbidity and age are established risk factors [19-21].

This study evaluates if family history has impact on the manifestation of hernia recurrence. We investigated patients having more than one recurrence where the primary and secondary hernia repair failed. Differences in the risk profile of primary and recurrent inguinal hernia patients might help to identify 'biological subgroups' of patients at risk. Referring to the European Hernia Society guidelines the identification of biological subgroups can be of importance to develop a tailored approach for hernia surgery[22].

## Methods

### Study design

Patients with more than 1 recurrence and age >18 years were included in the study. 214 cases were collected between January 1992 and February 2001 in the Surgical Department, University hospital, Aachen, Germany. Exclusion criteria were incisional hernia, death, no or one recurrence. The study was approved by the ethics committee of the University of Aachen and informed consent was obtained from all patients.

### Acquisition of data and selection of variables

Data were obtained by using a standardized questionnaire (Additional file 1). All interviews were done by one single research assistant. Data were collected on gender, age, smoking, comorbidity (constipation, COPD, coronary heart disease, diabetes, hypertension), medication and family history. Family history was defined as families with two or more affected parents, brothers, sisters, daughters and/or sons. Patients were subdivided into a group of patients with "no family history" and a group of patients with "family history". The incidence of family history was calculated and the impact of family history on the onset of primary hernia, the age at first recurrence and recurrence rates was analyzed. Recurrence rates were defined as reoperation rates for recurrent hernias. Results were adjusted to the distribution of risk factors in both subgroups.

### Statistics

Statistical analyses were carried out using the Statistical Package for the Social Sciences (SPSS 14.0, Chicago, USA). Data were organized according to subgroups with/without family history and according to risk factors. Data were tested by Student t test or Chi squared test when appropriate. The relative risk was estimated by calculating the odds ratio.

## Results

105 of 214 patients were dead or could not be contacted, 17 patients refused to participate and 17 patients had incisional hernias and were therefore excluded. 75 of these patients were eligible for the study.

33 of 75 analyzed inguinal hernia patients had affected relatives, and the incidence of family history was 44%. As expected, more male patients had inguinal hernias (67 male vs. 8 female patients) but distribution of gender (p = 0.27), smoking habits (p = 0.45), comorbidity and medication did not vary when subgroups with or without affected relatives were compared (Table 1).

**Table 1:** Distribution of risk factors in 74 patients with/without family history of hernia disease

Variable	Family history	Numbers of patients	P value	odds ratio
Male/female	no	39/3	0.27*	2.32
	yes	28/5		
Smoking	no	13	0.45	1.45
	yes	13		
Constipation	no	3	0.85	0.84
	yes	2		
COPD	no	6	0.78	0.82
	yes	4		
Diabetes	no	5	0.70	0.74
	yes	3		
Coronary heart disease	no	5	0.68	1.32
	yes	5		
Hypertension	no	16	0.67	0.81
	yes	11		
Medication	no	25	0.08	0.44
	yes	13		

* distribution of gender in patients with/without family history

Although age distribution was equal in both groups patients with family history had their primary hernia as well as their recurrence earlier. The onset of primary hernia was 41 years for patients with family history (range 18-64 years) and 48 years for patients without family history of hernia disease (range 18 -74 years, p = 0.04, table 2). Hernia recurrence occurred about 7 years earlier in patients with family history (46 versus 53 years p = 0.04, table 2).

**Table 2:** Impact of family history on disease manifestation

Variable	Family history	Age	p
Age (years)	no	63 (36-75)	0.37
	yes	61 (27-75)	
**Onset of primary hernia (years)**	no	**48 (18-74)**	**0.04**
	yes	**41 (18-65)**	
**Age at first recurrence (years)**	no	**53 (26-75)**	**0.04**
	yes	**46 (32-68)**	

Age is represented in mean years and age range.

**Figure 1:**
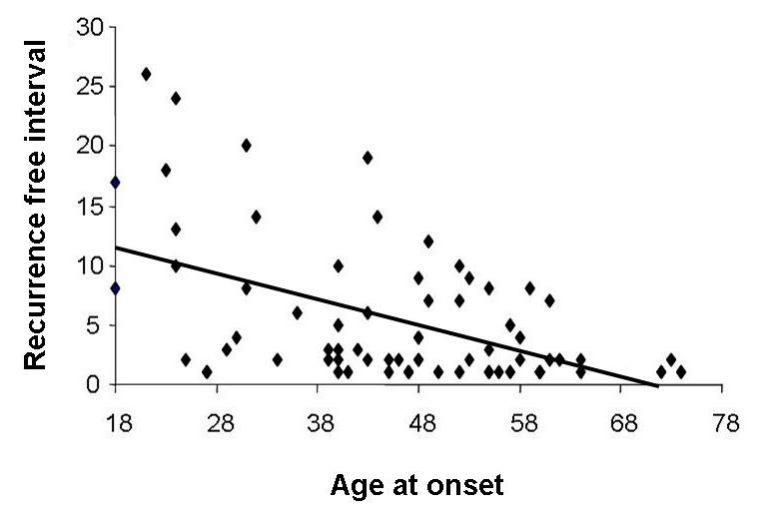
**Significant correlation between age of onset and recurrence-free interval (Pearsons correlation coefficient -0.53, p < 0.001)**.

A significant correlation could be detected between onset of primary hernia and the disease-free interval (Pearson's correlation coefficient r = -0.518, p = 0.01, Figure 1). The older the patients were at onset, the earlier they had a hernia recurrence.

Additionally, primary hernias occurred significantly earlier in smokers (p = 0.006) but not in patients with coronary heart disease or hypertension (Table 3).

**Table 3:** Impact of risk factors on disease manifestation

Variable		Age	p	Onset of primary hernia	p	Age at first recurrence	p
**Smoking**	no	63 (+/-12)	0.25	**47 (+/-6)**	**0.006**	50 (+/-7)	0.32
	yes	61(+/-11)		**44 (+/-4)**		48 (+/-6)	
**Coronary heart disease**	no	62 (+/-11)	0.09	45 (+/-15)	0.6	50 (+/-13)	0.71
	yes	68 (+/-9)		42 (+/-18)		48 (+/-15)	
**Hypertension**	no	61 (+/-12)	0.23	43 (+/-15)	0.29	49 (+/-13)	0.27
	yes	64 (+/-10)		47 (+/-15)		52 (+/-13)	

Data are represented in mean years +/- standard deviation.

Family history or smoking had no impact on the overall recurrence rate (p = 0.82 and p = 0.83, table 4). Coronary heart disease and hypertension were predictive factors for high recurrence rates (p = 0.001 and p = 0.03, table 4). These data correlated with anti-hypertensive medication and ACE inhibitors (data not shown).

**Table 4:** Impact of family history and risk factors on recurrence rates

Variable		Recurrence rate	p
Family history	no	4 (+/-2)	0.82
	yes	4 (+/+2)	
Smoking	no	4 (+/-2)	0.83
	yes	4 (+/-2)	
**Coronary heart disease**	no	**4 (+/-1)**	**0.001**
	yes	**6 (+/-3)**	
**Hypertension**	no	**4 (+/-1)**	**0.03**
	yes	**5 (+/-2)**	

Data are represented in mean number of recurrences +/- standard deviation.

## Discussion

The new finding of our study is that family history is not only a risk for primary hernias but also for hernia recurrence. In the present study 44% of the analyzed recurrent hernia patients have a family history of hernia disease. Lau et al. observed a rate of about 20% of patients with affected relatives developing primary inguinal hernias[23]. The importance of family history is reflected by a significantly earlier onset of primary as well as recurrent hernia disease. This study was based on the assumption that hernias have a genetic background. Several studies hint on collagen genes as candidate genes[18]. Collagens are very important for a sufficient wound healing. Family history as a risk factor for early recurrence would support the hypothesis that collagen gene or genes associated to collagen metabolism play a role for hernia disease.

Though the incidence of family history is considerably higher in recurrent hernia patients than in patients with primary hernias we could not detect any differences of recurrence rates between patients with or without family history. However, we have to consider an age-dependent recall bias as recurrence rates continuously increase with age. In our cohort more than 5 recurrences could be observed exclusively for patients that were older than 50 years and we have to expect a rise of recurrence rates for younger patients. Interestingly, recurrence rates were influenced by coronary heart disease and hypertension as well as by the corresponding medication. Regarding the fact that patients with coronary heart disease are significantly older, the increased recurrence rate can result from a prolonged observation period for these patients rather than from the coronary heart disease. However, coronary heart disease is known to be associated with disturbed collagen metabolism[24].

As expected hernia recurrence was influenced by patients' age. We found that the younger the patients were at onset the later they had a hernia recurrence. The age dependent manifestation of recurrence can be an argument for a tailored approach, especially if we think of complications due to mesh implantation.

The described risk factors are known to have impact on collagen regulation at different levels. Family history has to be correlated to a disturbed gene regulation whereas smoking induces a lack of ascorbic acid and hinders fibrillogenesis. The abnormal collagen expression in tissue samples of hernia patients results from the interactions between these factors. Comparing our results with those obtained from literature it becomes obvious that family history and age are risk factors for both, primary and recurrent inguinal hernia disease. Indeed, our study is limited to 75 patients with re-recurrent hernias. But in contrast to patients with primary hernias, smoking could not be identified as a risk factos for re-recurrent hernias. As a consequence different molecular pathways have to be assumed and especially genetic analyses have to be performed in carefully defined subsets of patients. Though an altered collagen composition is known, therapeutic approaches for hernias are still restricted to reinforcement of the abdominal wall. The identification of susceptible genes could permit causative treatment options as realized for the treatment of aortic aneurysm by distinctive blockage of gene transcription in animal models[25]. Meshes can be used as carrier systems for potential drug treatment. Current research may focus on the identification of patients at risk for recurrence or the prevention of hernia recurrence by improving wound healing.

## Conclusion

In summary, our data reveal an increased incidence of family history for recurrent hernia patients when compared with primary hernia patients. Patients with a family history have their primary hernias as well as their recurrence at younger age then patients without a family history. Though recurrent hernia has to be regarded as a disease caused by multiple factors, a family history may be considered as a criterion to identify the risk for recurrence before the primary operation.

## Competing interests

The authors declare that they have no competing interests.

## Authors' contributions

PLJ carried out the design of the study, the data analysis and drafted the manuscript. KJ conceived of the study, and participated in its design and coordination and drafted the manuscript. MJ participated in the data analysis and helped to draft the manuscript. UK participated in the design of the study and performed the statistical analysis. All authors read and approved the final manuscript.

## Pre-publication history

The pre-publication history for this paper can be accessed here:

http://www.biomedcentral.com/1471-2482/9/18/prepub

## Supplementary Material

Additional file 1 Standardized questionnaire used in this study. Click here for file
