# Supplementary material for: Risk factors for early recurrence after inguinal hernia repair
Source: BMC Surg. 2009 Dec 9;9:18. doi: 10.1186/1471-2482-9-18 (PMC2795732; doi:10.1186/1471-2482-9-18)
Supplement: Additional file 1 — Standardized questionnaire used in this study. [file 1471-2482-9-18-S1.DOC]

# CHIRURGISCHE UNIVERSITÄTSKLINIK UND POLIKLINIK

**DER RWTH AACHEN**

Direktor: Universitätsprofessor Dr. med. Dr. h.c. V. Schumpelick

Chirurgische Universitätsklinik u. Poliklinik der RWTH Aachen Telefon: (0241)8089500/501

Pauwelsstraße 30, D -52074 Aachen Telefax: (0241)8888417

Öffentliche Verkehrsmittel:

Bus, Linien: SB 1,3,5,33,45,70

Auskunft erteilt:______________________

Zimmer- Nr.:_________________________

**Bitte mein Zeichen bei Antwort angeben!**

Zeichen und Tag Ihres Schreibens Mein Zeichen Datum:

29.08.26

**Betr.: Befragung von Hernien- Rezidiv- Patienten**

Sehr geehrter (Name)

Die wiederholte Entstehung von Leisten- und Narben- Brüchen (rezidivierende Hernien) und auch das gehäufte Auftreten von Hernien in der Familie der Betroffenen lassen eine mögliche Vererbung dieser Erkrankung vermuten.

In einer breit angelegten wissenschaftlichen Studie versuchen wir Familien mit einem erhöhten Risiko für das Auftreten von Brüchen zu finden, um aus den gewonnen Erkenntnissen Vorsorge- Konzepte zu entwickeln.

Aufgrund der auch bei Ihnen wiederholt aufgetretenen Hernien ist uns bei unserem Forschungsprojekt Ihre Hilfe besonders wichtig. Daher möchten wir Sie bitten, den beigefügten Fragebogen nach bestem Wissen auszufüllen und diesen mit Hilfe des vorfrankierten Rückumschlages in den nächsten 14 Tagen an die oben angegebene Adresse zurückzusenden (Stichwort: Genetikstudie)

Bitte lassen Sie sich vom Umfang des Fragebogens nicht entmutigen: die Fragen werden durch die Angaben zu den verschiedenen Personenkreisen – zu Ihren Eltern, zu Geschwistern Ihrer Eltern, zu eigenen Geschwistern und zu eigenen Kindern – sich mehrmals wiederholen bzw. teilweise sogar ganz entfallen.

Selbstverständlich werden die aus der Befragung gewonnenen Erkenntnisse anonym behandelt und ausschließlich für wissenschaftliche Zwecke verwendet.

Wir bedanken uns für Ihre engagierte Mitarbeit und stehen Ihnen für weitere Informationen gern zur Verfügung.

E. Bergert OA Dr. med. U. Klinge

## Fragebogen

**Für die klinische Studie zur Frage des genetischen Einflusses auf die Entstehung von Leisten- und Narben- Hernien**

**Chirurgische Klinik der RWTH Aachen**

### **Direktor: Univ. Prof. Dr. med. Dr. h.c. V. Schumpelick**

# EINVERSTÄNDNISERKLÄRUNG

Mit der anonymen Verwendung der Daten im Rahmen einer wissenschaftlichen Studie bin ich einverstanden.

Name:______________________________________________________________

Geburtsdatum:_______________________________________________________

Ort:__________________ Datum:_________________________

Unterschrift:________________________________________________________

#### Inhaltsverzeichnis

**Seite**

A) Angaben zu Ihrer Person 3

B) Angaben zu Ihren Eltern 8

C) Angaben zu Geschwistern Ihrer Mutter 12

D) Angaben zu Geschwistern Ihres Vaters 16

E) Angaben zu eigenen Geschwistern 21

F) Angaben zu eigenen Kindern 25

**Bitte lassen Sie sich vom Umfang des Fragebogens nicht entmutigen:**

**die Fragen werden durch die gewünschten Angaben zu den verschiedenen Personenkreisen**

**– zu Ihren Eltern, zu Geschwistern Ihrer Eltern, zu eigenen Geschwistern und zu eigenen Kindern –**

**sich mehrmals wiederholen bzw. teilweise sogar ganz entfallen.**

A

**Angaben zu Ihrer Person**

| **Name:** |  |
| --- | --- |
| **Vorname:** |  |
| **Geschlecht:** | ** weiblich  männlich** |
| **Geburts-datum:** |  |
| **Wohnort:** |  |
| **Straße:** |  |
| **Telefon-nummer:** |  |
| **Beruf:** |  |
| **Hausarzt:** |  |
| **Ort:** |  |
| **Straße:** |  |
| **Telefon-nummer:** |  |

Bei Ihnen wurde in unserer Klinik ein **Leistenbruch** operiert.

| **Nr.** | **Frage:** | **Antwort :** |
| --- | --- | --- |
| **A1** | Auf welcher **Seite** befand sich der Leistenbruch ? | ** links  beide Seiten**  ** rechts** |
| **A2** | Wenn der Leistenbruch **rechts** auftrat, wiederholte sich dieser, so daß eine weitere Operation erforderlich war? | ** ja unbekannt**  ** nein** |
| **A3** | Wenn ja, **wie oft** und **wann** trat der Leistenbruch insgesamt auf der rechten Seite auf? |  |
| **A**  **4** | Wenn der Leistenbruch **links** auftrat, wiederholte sich dieser, so daß eine weitere Operation erforderlich war? | ** ja unbekannt**  ** nein** |
| **A**  **5** | Wenn ja, **wie oft** und **wann** trat insgesamt der Leistenbruch auf der linken Seite auf? |  |
| **A6** | Bei Operationen kann durch Schnitte der Haut und durch den anschließenden Wundverschluß das **Gewebe geschwächt** werden. So kann es im Bereich der entstehenden Narbe zu einem sog. **Narbenbruch** kommen. **Wurden bei Ihnen während Operationen größere Hautschnitte gesetzt?** (Hier bitte nicht ihre Leistenbruch – Operationen anführen)  [wenn nicht weiter mit Frage A11] | ** ja**  ** nein**  **unbekannt** |

| **A7** | Wenn ja, um **welche** Operationen handelte es sich? z.B.. Gallen – Operation oder Blinddarmoperation, Kaiserschnitt etc.  Hier bitte nicht ihre Leistenbruch – Operationen anführen | **1.**  **2.**  **3.** |
| --- | --- | --- |
| **A8** | Geben Sie bitte die ungefähren **Daten** der von Ihnen angeführten Operationen an! | **1.**  **2.**  **3.** |
| **A9** | Trat an den von Ihnen angeführten Operationsnarben schon einmal ein **Narbenbruch** auf? | ** ja**  ** nein**  **unbekannt** |
| **A10** | Wenn ja, **wie oft** und **wann** trat der Bruch an den jeweiligen Narben auf? | **an der 1. Narbe:**  **an der 2.Narbe:**  **an der 3.Narbe:** |

**Die Erhöhung des Bauchinnendrucks ist erwiesenermaßen auch ein Auslöser von Leisten- und Narben-Hernien. Darum befragen wir Sie im folgenden nach verschiedenen Erkrankungen, die den Bauchinnendruck beeinflussen!**

| **Nr.** | **Erkrankung** | **Antwort** | | **Datum der Diagnosestellung** |
| --- | --- | --- | --- | --- |
| **A11** | Litten Sie über längere Zeit an einer **chronischen Verstopfung** (Obstipation), die von einem Arzt festgestellt und medikamentös behandelt wurde? | ** ja unbekannt**  ** nein** | |  |
| **A12** | Wenn eine dauerhafte Verstopfung festgestellt wurde, **wie lange** dauerte die Verstopfung ungefähr an ? |  | | |
| **A13** | Litten Sie über einen längeren Zeitraum an einer **chronischen Bronchitis**, die ärztlich nachgewiesen und durch eine Arzt medikamentös behandelt wurde? | ** ja unbekannt**  ** nein** | |  |
| **A14** | **Wie lange** dauerte die chronische  Bronchitis an ? |  | | |
| **A15** | **Wann** stellte ein Arzt erstmals eine so langwierige Bronchitis bei Ihnen fest? |  | | |
| **A16** | Stellte man bei Ihnen eine **Zuckererkrankung** (Diabetes) fest? | ** ja unbekannt**  ** nein** | |  |
| **A17** | Wenn ja , sind Sie aufgrund dieser Zuckererkrankung **insulinpflichtig oder nicht?** | ** ja unbekannt**  ** nein** | | |
| **Nr.** | **Erkrankung** | **Antwort** | | **Datum der Diagnosestellung** |
| **A18** | Stellte ein Arzt eine **Koronare Herzerkrankung** bei Ihnen fest? | ** ja unbekannt**  ** nein** | |  |
| **A19** | Leiden Sie an **Bluthochdruck** (Hypertonie), der medikamentös eingestellt ist? | ** ja unbekannt**  ** nein** | |  |
| **A20** | Bei **Männern**: wurde bei Ihnen eine **Vergrößerung der Prostata** durch einen Arzt festgestellt ? | ** ja unbekannt**  ** nein** | |  |
| **A21** | Stellte man bei Ihnen einen **bösartigen Tumor** **in der Bauchregion** fest? | ** ja unbekannt**  ** nein** | |  |
| **A22** | Wenn ja, kennen Sie den **Namen des Tumors**? Bitte geben Sie diesen an oder beschreiben Sie den Tumor, wenn Sie können, näher! **Wann** ist dieser aufgetreten? **Wurde** **operiert ?** |  | | |
| **A23** | Stellte ein Arzt bei Ihnen **sonstige** **Erkrankungen** fest? | ** ja unbekannt**  ** nein** | | |
| **A24** | Wenn ja, geben Sie bitte den **Namen** der Erkrankung und das entsprechende erstmalige Feststellungsdatum an! Falls der Platz nicht ausreicht, bitte nutzen Sie die Rückseite! | **1.**  **2.**  **3.** | | **4.**  **5.**  **6.** |
| **A25** | Bitte geben Sie die **Namen** aller ärztlich verschriebenen Medikamente, die Sie einnehmen müssen und das entsprechenden **Datum der erstmaligen Verschreibung** an. Falls der Platz unzureichend ist, nutzen Sie bitte auch die Rückseite! | **1.**  **2.**  **3.** | | |
| **A26** | Haben Sie in der Zeit bis zur 1. Wiederholung Ihres Bruches regelmäßig **geraucht**? [wenn Sie nicht geraucht haben, weiter mit Frage A32] | | **ja**  ** nein, ich habe noch nie regelmäßig geraucht !** | |
| **A27** | Wenn Sie bis dahin regelmäßig geraucht haben, **wieviel Jahre** haben Sie bis dahin geraucht? | |  | |
| **A28** | Wenn Sie bis dahin regelmäßig geraucht haben, **wieviel** Zigaretten pro Tag ? | |  | |
| **A29** | Bei *Zigarren, Zigarillos oder Pfeifenrauchern*: **wieviel** pro Tag? | |  | |
| **A30** | Rauchten Sie **zu Anfang weniger?** Wenn ja, mit **wieviel** Zigaretten pro Tag haben Sie begonnen? | | **ja, zu Anfang rauchte ich nur:_______________**  **nein, ich rauchte immer soviel wie zuletzt** | |
| **A31** | Bei *Zigarren, Zigarillos oder Pfeifenrauchern*: Rauchten Sie **zu Anfang weniger**? Wenn ja, mit **wieviel** Zigarren, Zigarillos oder  Pfeifen pro Tag haben Sie begonnen? | | **ja, zu Anfang rauchte ich nur:_______________**  **nein, ich rauchte immer soviel wie zuletzt** | |

**A32) Bestimmt haben auch Sie sich Gedanken gemacht , warum die Brüche bei Ihnen wiederholt auftraten. Bitte lassen Sie uns an Ihren Überlegungen teilhaben und schreiben Sie uns Ihre Erklärungen hier und umseitig kurz auf.**

B

**Angaben zu Ihren Eltern**

**Um eine genetische Veranlagung nachweisen zu können, sind Angaben zu Ihrer Familie erforderlich. Beantworten Sie bitte die folgenden Fragen zu Ihren Familienangehörigen, soweit Ihnen die Angaben bekannt sind. Steht Ihnen die gewünschte Information nicht zur Verfügung , kreuzen Sie bitte „unbekannt“ an oder tragen Sie ein „?“ in das leere Kästchen ein!!**

| **Nr.** | Frage | Mutter | Vater |
| --- | --- | --- | --- |
| **B1** | **Geburtsdatum** Ihrer Mutter/  Ihres Vaters ? |  |  |
| **B2** | Wurde bei Ihrer Mutter / Ihrem Vater jemals eine **Bauchoperation** durchgeführt? [wenn nicht, weiter mit Frage B7] | ** ja unbekannt**  ** nein** | ** ja unbekannt**  ** nein** |
| **B3** | Wenn ja, um **welche** **Operation** handelte es sich? | **1.**  **2.**  **3.** | **1.**  **2.**  **3.** |
| **B4** | Geben Sie bitte die ungefähren **Daten** der von Ihnen angeführten Operationen bei Ihrer Mutter / Ihrem Vater an! | **1.**  **2.**  **3.** | **1.**  **2.**  **3.** |
| **B5** | Trat an den von Ihnen angeführten Operationsnarben bei Ihrer Mutter / Ihrem Vater schon einmal ein **Narbenbruch** auf?  [wenn nicht weiter mit Frage 7] | ** ja unbekannt**  ** nein** | ** ja unbekannt**  ** nein** |
| **B6** | Wenn ja, **wie oft** und **wann** trat der Bruch an den jeweiligen Narben Ihrer Mutter / Ihres Vaters auf? | **1.Narbe:**  **2.Narbe:**  **3.Narbe:** | **1.Narbe:**  **2.Narbe:**  **3.Narbe:** |
| **B7** | Trat bei Ihrer Mutter/ Ihrem Vater schon einmal ein **Leistenbruch** auf?  [wenn nicht weiter mit Frage B13] | ** ja unbekannt**  ** nein** | ** ja unbekannt**  ** nein** |
| **B8** | Wenn ja, auf welcher **Seite**?  [wenn nur links, weiter mit Frage B11, wenn nur rechts oder beidseitig weiter mit Frage B9] | ** links  beide Seiten**  ** rechts** | ** links  beide Seiten**  ** rechts** |
| **B9** | Wenn der **Leistenbruch rechts** auftrat, wiederholte sich dieser bei Ihrer Mutter/ Ihrem Vater? | ** ja unbekannt**  ** nein** | ** ja unbekannt**  ** nein** |
| **B10** | Wenn ja, **wie oft** und **wann** trat der Leistenbruch insgesamt auf der rechten Seite bei Ihrer Mutter / Ihrem Vater auf? |  |  |
| **B11** | Wenn der **Leistenbruch links** auftrat, wiederholte sich dieser bei Ihrer Mutter/ Ihrem Vater? | ** ja unbekannt**  ** nein** | ** ja unbekannt**  ** nein** |
| **B12** | Wenn ja, **wie oft** und **wann** trat insgesamt der Leistenbruch auf der linken Seite Ihrer Mutter / Ihrem Vater auf? |  |  |

**Nur wenn bei Ihrer Mutter / Ihrem Vater ein Narben- oder Leistenbruch wiederholt auftrat, ist es notwendig auch die folgenden Fragen zu beantworten. Wenn bei Ihren Eltern kein Bruch auftrat, bitte weiter auf Seite 12 !**

| **Nr.** | **Erkrankung** | Mutter | **Datum der Diagnose-**  **stellung** | **Vater** | **Datum der Diagnose-**  **stellung** |
| --- | --- | --- | --- | --- | --- |
| **B13** | Litt Ihre Mutter/ Ihr Vater über längere Zeit an einer **chronischen Verstopfung** (Obstipation) , die von einem Arzt festgestellt und medikamentös behandelt wurde? | ** ja**  ** nein**  **unbekannt** |  | ** ja**  ** nein**  **unbekannt** |  |
| **B14** | Wenn eine dauerhafte Verstopfung festgestellt wurde, **wie lange** dauerte die Verstopfung bei Ihrer Mutter/ Ihrem Vater ungefähr an ? |  | |  | |
| **B15** | Litt Ihre Mutter/ Ihr Vater über einen längeren Zeitraum an einer **chronischen Bronchitis**, die ärztlich nachgewiesen und durch eine Arzt medikamentös behandelt wurde? | ** ja**  ** nein**  **unbekannt** |  | ** ja**  ** nein**  **unbekannt** |  |
| **B16** | **Wie lange** dauerte die chronische  Bronchitis bei Ihrer Mutter /Ihrem Vater an ? |  | |  | |
| **B17** | **Wann** stellte ein Arzt erstmals eine so langwierige Bronchitis bei Ihrer Mutter /Ihrem Vater fest? |  | |  | |
| **B18** | Stellte man bei Ihrer Mutter/ Ihrem Vater eine **Zuckererkrankung** (Diabetes) fest? | ** ja**  ** nein**  **unbekannt** |  | ** ja**  ** nein**  **unbekannt** |  |
| **B19** | Wenn ja , ist Ihre Mutter / Ihr Vater aufgrund dieser Zuckererkrankung **insulinpflichtig oder nicht**? | ** ja**  ** nein**  **unbekannt** | | ** ja**  ** nein**  **unbekannt** | |
| **B20** | Stellte ein Arzt eine **Koronare Herzerkrankung** bei Ihrer Mutter/ Ihrem Vater fest? | ** ja**  ** nein**  **unbekannt** |  | ** ja**  ** nein**  **unbekannt** |  |
| **B21** | Leidet / Litt Ihre Mutter / Ihr Vater an **Bluthochdruck** (Hypertonie), der medikamentös eingestellt ist? | ** ja**  ** nein**  **unbekannt** |  | ** ja**  ** nein**  **unbekannt** |  |
| **B22** | Wurde bei Ihrem Vater eine **Vergrößerung der Prostata** durch einen Arzt festgestellt ? |  | | ** ja**  ** nein**  **unbekannt** |  |
| **Nr.** | **Erkrankung** | Mutter | **Datum der Diagnose-**  **stellung** | **Vater** | **Datum der Diagnose-**  **stellung** |
| **B23** | Stellte man bei Ihrer Mutter/Ihrem Vater einen **bösartigen Tumor** in der Bauchregion fest? | ** ja**  ** nein**  **unbekannt** |  | ** ja**  ** nein**  **unbekannt** |  |
| **B24** | Wenn ja, kennen Sie den **Namen des Tumors**? Bitte geben Sie diesen an oder beschreiben Sie den Tumor, wenn Sie können, näher! **Wann** ist dieser aufgetreten? **Wurde operiert ?** |  |  | ** ja**  ** nein**  **unbekannt** |  |
| **B25** | Stellte ein Arzt bei Ihrer Mutter /Ihrem Vater **sonstige Erkrankungen** fest? | ** ja**  ** nein**  **unbekannt** |  | ** ja**  ** nein**  **unbekannt** |  |
| **B26** | Wenn ja, schreiben Sie bitte den **Namen der Erkrankung** und das entsprechende erstmalige **Feststellungsdatum** hier auf! | | | | |

C

**Geschwister Ihrer Mutter**

**Falls Ihre Mutter keine Geschwister hat, setzen Sie bitte auf der Seite 16 mit den Angaben zu den Geschwistern Ihres Vaters fort. Sicherlich sind Ihnen manche Daten nicht bekannt! Versuchen Sie bitte trotzdem die Angaben zu machen, die Ihnen zur Verfügung stehen! Ansonsten kreuzen Sie bitte „unbekannt“ an oder tragen Sie ein „?“ in das Kästchen ein!**

**C1) Wie viele Geschwister hat Ihre Mutter (bitte auch Verstorbene angeben)?____________________**

| **Nr.** | **Frage** | **1.**  **Schwester/**  **Bruder Ihrer Mutter** | **2.**  **Schwester/**  **Bruder Ihrer Mutter** | **3.**  **Schwester/**  **Bruder Ihrer Mutter** | **4.**  **Schwester/**  **Bruder Ihrer Mutter** | **5.**  **Schwester/**  **Bruder Ihrer Mutter** |
| --- | --- | --- | --- | --- | --- | --- |
| **C2** | **Geburtsdatum** der Geschwister  Ihrer Mutter ? |  |  |  |  |  |
| **C3** | **Geschlecht** der Geschwister Ihrer Mutter? | **männlich**  **weiblich** | **männlich**  **weiblich** | **männlich**  **weiblich** | **männlich**  **weiblich** | **männlich**  **weiblich** |
| **C4** | Wurde bei Geschwistern Ihrer Mutter jemals eine **Bauchoperation** durchgeführt? [wenn nicht, weiter mit Frage C8] | **ja**  **nein**  **unbekannt** | **ja**  **nein**  **unbekannt** | **ja**  **nein**  **unbekannt** | **ja**  **nein**  **unbekannt** | **ja**  **nein**  **unbekannt** |
| **C5** | Wenn ja, um **welche** Operation handelte es sich? | **1.**  **2.**  **3.** | **1.**  **2.**  **3.** | **1.**  **2.**  **3.** | **1.**  **2.**  **3.** | **1.**  **2.**  **3.** |
| **C6** | Trat an den von Ihnen angeführten Operationsnarben bei Geschwistern Ihrer Mutter schon einmal ein **Narbenbruch** auf? [wenn nicht weiter mit Frage C8] | **ja**  **nein**  **unbekannt** | **ja**  **nein**  **unbekannt** | **ja**  **nein**  **unbekannt** | **ja**  **nein**  **unbekannt** | **ja**  **nein**  **unbekannt** |
| **C7** | Wenn ja, **wie oft** und **wann** trat der Bruch an den jeweiligen Narben der Geschwister Ihrer Mutter auf? | **1.Narbe:**  **2.Narbe:**  **3.Narbe:** | **1.Narbe:**  **2.Narbe:**  **3.Narbe:** | **1.Narbe:**  **2.Narbe:**  **3.Narbe:** | **1.Narbe:**  **2.Narbe:**  **3.Narbe:** | **1.Narbe:**  **2.Narbe:**  **3.Narbe:** |
| **C8** | Trat bei Geschwistern Ihrer Mutter schon einmal ein **Leistenbruch** auf?  [wenn nicht weiter mit Frage C14] | **ja**  **nein**  **unbekannt** | **ja**  **nein**  **unbekannt** | **ja**  **nein**  **unbekannt** | **ja**  **nein**  **unbekannt** | **ja**  **nein**  **unbekannt** |
| **C9** | Wenn ja, auf welcher **Seite**?  [wenn nur links, weiter mit Frage C12, wenn nur rechts oder beidseitig weiter mit Frage C10] | ** links**  ** rechts**  ** beide Seiten** | ** links**  ** rechts**  ** beide Seiten** | ** links**  ** rechts**  ** beide Seiten** | ** links**  ** rechts**  ** beide Seiten** | ** links**  ** rechts**  ** beide Seiten** |
| **C10** | Wenn der **Leistenbruch rechts** auftrat, wiederholte sich dieser bei Geschwistern Ihrer Mutter ? | **ja**  **nein**  **unbekannt** | **ja**  **nein**  **unbekannt** | **ja**  **nein**  **unbekannt** | **ja**  **nein**  **unbekannt** | **ja**  **nein**  **unbekannt** |

|  | **Frage** | **1.**  **Schwester/**  **Bruder Ihrer Mutter** | **2.**  **Schwester/**  **Bruder Ihrer Mutter** | **3.**  **Schwester/**  **Bruder Ihrer Mutter** | **4.**  **Schwester/**  **Bruder Ihrer Mutter** | **5.**  **Schwester/**  **Bruder Ihrer Mutter** |
| --- | --- | --- | --- | --- | --- | --- |
| **C11** | Wenn ja, **wie oft** und **wann** trat der Leistenbruch insgesamt auf der rechten Seite bei Geschwistern Ihrer Mutter auf? |  |  |  |  |  |
| **C12** | Wenn der **Leistenbruch links** auftrat, wiederholte sich dieser bei Geschwistern Ihrer Mutter? | **ja**  **nein**  **unbekannt** | **ja**  **nein**  **unbekannt** | **ja**  **nein**  **unbekannt** | **ja**  **nein**  **unbekannt** | **ja**  **nein**  **unbekannt** |
| **C13** | Wenn ja, **wie oft** und **wann** trat insgesamt der Leistenbruch auf der linken Seite bei Geschwistern Ihrer Mutter auf? |  |  |  |  |  |

**Nur wenn bei Geschwistern Ihrer Mutter ein Narben- oder Leistenbruch wiederholt auftrat, ist es notwendig auch die folgenden Fragen zu beantworten. Ansonsten bitte weiter auf Seite 16!**

| **Nr.** | **Erkrankung** | **1.**  **Schwester/**  **Bruder Ihrer Mutter** | **2.**  **Schwester/**  **Bruder Ihrer Mutter** | **3.**  **Schwester/**  **Bruder Ihrer Mutter** | **4.**  **Schwester/**  **Bruder Ihrer Mutter** | **5.**  **Schwester/**  **Bruder Ihrer Mutter** |
| --- | --- | --- | --- | --- | --- | --- |
| **C14** | Litten Geschwister Ihrer Mutter über längere Zeit an einer **chronischen Verstopfung** (Obstipation) , die von einem Arzt festgestellt und medikamentös behandelt wurde? | **ja**  **nein**  **unbekannt** | **ja**  **nein**  **unbekannt** | **ja**  **nein**  **unbekannt** | **ja**  **nein**  **unbekannt** | **ja**  **nein**  **unbekannt** |
| **C15** | Wenn eine dauerhafte Verstopfung festgestellt wurde**, wie lange** dauerte die Verstopfung bei Geschwistern Ihrer Mutter/ ungefähr an? |  |  |  |  |  |
| **C16** | Litten Geschwister Ihrer Mutter über einen längeren Zeitraum an einer **chronischen Bronchitis**, die ärztlich nachgewiesen und durch eine Arzt medikamentös behandelt wurde? | **ja**  **nein**  **unbekannt** | **ja**  **nein**  **unbekannt** | **ja**  **nein**  **unbekannt** | **ja**  **nein**  **unbekannt** | **ja**  **nein**  **unbekannt** |
| **C17** | **Wie lange** dauerte die chronische  Bronchitis bei Geschwistern Ihrer Mutter an? |  |  |  |  |  |
| **C18** | **Wann** stellte ein Arzt erstmals eine so langwierige Bronchitis bei Geschwistern Ihrer Mutter fest? |  |  |  |  |  |
| **C19** | Stellte man bei Geschwistern Ihrer Mutter eine **Zuckererkrankung** (Diabetes) fest? | **ja**  **nein**  **unbekannt** | **ja**  **nein**  **unbekannt** | **ja**  **nein**  **unbekannt** | **ja**  **nein**  **unbekannt** | **ja**  **nein**  **unbekannt** |
| **C20** | Wenn ja , sind Geschwister Ihrer Mutter aufgrund dieser Zuckererkrankung **insulinpflichtig oder nicht?** | **ja**  **nein**  **unbekannt** | **ja**  **nein**  **unbekannt** | **ja**  **nein**  **unbekannt** | **ja**  **nein**  **unbekannt** | **ja**  **nein**  **unbekannt** |
| **Nr.** | **Erkrankung** | **1.**  **Schwester/**  **Bruder Ihrer Mutter** | **2.**  **Schwester/**  **Bruder Ihrer Mutter** | **3.**  **Schwester/**  **Bruder Ihrer Mutter** | **4.**  **Schwester/**  **Bruder Ihrer Mutter** | **5.**  **Schwester/**  **Bruder Ihrer Mutter** |
| **C21** | Geben Sie bitte, wenn Sie können, das **Datum der Erstfeststellung der Diabetes** durch einen Arzt an! |  |  |  |  |  |
| **C22** | Stellte ein Arzt eine **Koronare Herzerkrankung** bei Geschwistern Ihrer Mutter fest? | **ja**  **nein**  **unbekannt** | **ja**  **nein**  **unbekannt** | **ja**  **nein**  **unbekannt** | **ja**  **nein**  **unbekannt** | **ja**  **nein**  **unbekannt** |
| **C23** | Geben Sie bitte, wenn Sie können, das **Datum der Erstfeststellung der koronaren Herzerkrankung** durch einen Arzt an! |  |  |  |  |  |
| **C24** | Leiden Geschwister Ihrer Mutter an **Bluthochdruck** (Hypertonie), der medikamentös eingestellt ist? | **ja**  **nein**  **unbekannt** | **ja**  **nein**  **unbekannt** | **ja**  **nein**  **unbekannt** | **ja**  **nein**  **unbekannt** | **ja**  **nein**  **unbekannt** |
| **C25** | Geben Sie bitte, wenn Sie können, das **Datum der Erstfeststellung des Bluthochdrucks** durch einen Arzt an! |  |  |  |  |  |
| **C26** | Wurde bei Brüdern Ihrer Mutter eine **Vergrößerung der Prostata** durch einen Arzt festgestellt ? | **ja**  **nein**  **unbekannt** | **ja**  **nein**  **unbekannt** | **ja**  **nein**  **unbekannt** | **ja**  **nein**  **unbekannt** | **ja**  **nein**  **unbekannt** |
| **C27** | Geben Sie bitte, wenn Sie können, das **Datum der Erstfeststellung der Prostatavergrößerung** durch einen Arzt an! |  |  |  |  |  |
| **C28** | Stellte man bei Geschwistern Ihrer Mutter einen **bösartigen Tumor in der Bauchregion** fest? | **ja**  **nein**  **unbekannt** | **ja**  **nein**  **unbekannt** | **ja**  **nein**  **unbekannt** | **ja**  **nein**  **unbekannt** | **ja**  **nein**  **unbekannt** |
| **C29** | Wenn ja, kennen Sie den **Namen des Tumors**? Bitte geben Sie diesen an oder beschreiben Sie den Tumor, wenn Sie können, näher! **Wurde operiert?** |  |  |  |  |  |
| **C30** | Geben Sie bitte das **ungefähre Datum der ersten ärztlichen Feststellung des bösartigen Tumors** an ! |  |  |  |  |  |
| **C31**  **C32** | Stellte ein Arzt bei Geschwistern Ihrer Mutter **sonstige Erkrankungen** fest?  Wenn ja, schreiben Sie bitte den Namen der Erkrankung und das entsprechende erstmalige Feststellungsdatum bei Geschwistern Ihrer Mutter umseitig auf! | **ja**  **nein**  **unbekannt** | **ja**  **nein**  **unbekannt** | **ja**  **nein**  **unbekannt** | **ja**  **nein**  **unbekannt** | **ja**  **nein**  **unbekannt** |

D

**Geschwister Ihres Vaters**

**Falls Ihr Vater keine Geschwister hat, setzen Sie bitte auf der Seite 21 mit den Angaben zu den eigenen Geschwistern fort. Sicherlich sind Ihnen manche Daten der Geschwister Ihres Vaters nicht bekannt! Versuchen Sie bitte trotzdem die Angaben zu machen, die Ihnen zur Verfügung stehen! Ansonsten kreuzen Sie bitte „unbekannt“ an oder tragen Sie ein „?“ in das Kästchen ein!**

**D1) Wie viele Geschwister hat Ihr Vater (bitte auch Verstorbene angeben) ? _________________**

| **Nr.** | **Frage** | **1.**  **Schwester/**  **Bruder Ihres Vaters** | **2.**  **Schwester/**  **Bruder Ihres Vaters** | **3.**  **Schwester/**  **Bruder Ihres Vaters** | **4.**  **Schwester/**  **Bruder Ihres Vaters** | **5.**  **Schwester/**  **Bruder Ihres Vaters** |
| --- | --- | --- | --- | --- | --- | --- |
| **D2** | **Geburtsdatum** der Geschwister Ihres Vaters? |  |  |  |  |  |
| **D3** | **Geschlecht** der Geschwister Ihres Vaters? | **männlich**  **weiblich** | **männlich**  **weiblich** | **männlich**  **weiblich** | **männlich**  **weiblich** | **männlich**  **weiblich** |
| **D4** | Wurde bei Geschwistern Ihres Vaters jemals eine **Bauchoperation** durchgeführt? [wenn nicht, weiter mit Frage D8] | **ja**  **nein**  **unbekannt** | **ja**  **nein**  **unbekannt** | **ja**  **nein**  **unbekannt** | **ja**  **nein**  **unbekannt** | **ja**  **nein**  **unbekannt** |
| **D5** | Wenn ja, um **welche** Operation handelte es sich? | **1.**  **2.**  **3.** | **1.**  **2.**  **3.** | **1.**  **2.**  **3.** | **1.**  **2.**  **3.** | **1.**  **2.**  **3.** |
| **D6** | Trat an den von Ihnen angeführten Operationsnarben bei Geschwistern Ihres Vaters schon einmal ein **Narbenbruch** auf? [wenn nicht weiter mit Frage D8] | **ja**  **nein**  **unbekannt** | **ja**  **nein**  **unbekannt** | **ja**  **nein**  **unbekannt** | **ja**  **nein**  **unbekannt** | **ja**  **nein**  **unbekannt** |
| **D7** | Wenn ja, **wie oft** und **wann** trat der Bruch an den jeweiligen Narben der Geschwister Ihres Vaters auf? | **1.Narbe:**  **2.Narbe:**  **3.Narbe:** | **1.Narbe:**  **2.Narbe:**  **3.Narbe:** | **1.Narbe:**  **2.Narbe:**  **3.Narbe:** | **1.Narbe:**  **2.Narbe:**  **3.Narbe:** | **1.Narbe:**  **2.Narbe:**  **3.Narbe:** |
| **D8** | Trat bei Geschwistern Ihres Vaters schon einmal ein **Leistenbruch** auf?  [wenn nicht weiter mit Frage D14] | **ja**  **nein**  **unbekannt** | **ja**  **nein**  **unbekannt** | **ja**  **nein**  **unbekannt** | **ja**  **nein**  **unbekannt** | **ja**  **nein**  **unbekannt** |
| **D9** | Wenn ja, auf welcher **Seite**?  [wenn nur links, weiter mit Frage D12, wenn nur rechts oder beidseitig weiter mit Frage D10] | ** links**  ** rechts**  ** beide Seiten** | ** links**  ** rechts**  ** beide Seiten** | ** links**  ** rechts**  ** beide Seiten** | ** links**  ** rechts**  ** beide Seiten** | ** links**  ** rechts**  ** beide Seiten** |
| **D10** | Wenn der **Leistenbruch rechts** auftrat, wiederholte sich dieser bei Geschwistern Ihres Vaters? | **ja**  **nein**  **unbekannt** | **ja**  **nein**  **unbekannt** | **ja**  **nein**  **unbekannt** | **ja**  **nein**  **unbekannt** | **ja**  **nein**  **unbekannt** |

| **Nr.** | **Frage** | **1.**  **Schwester/**  **Bruder Ihres Vaters** | **2.**  **Schwester/**  **Bruder Ihres Vaters** | **3.**  **Schwester/**  **Bruder Ihres Vaters** | **4.**  **Schwester/**  **Bruder Ihres Vaters** | **5.**  **Schwester/**  **Bruder Ihres Vaters** |
| --- | --- | --- | --- | --- | --- | --- |
| **D11** | Wenn ja, **wie oft** und **wann** trat der Leistenbruch insgesamt auf der rechten Seite bei Geschwistern Ihres Vaters auf? |  |  |  |  |  |
| **D12** | Wenn der **Leistenbruch links** auftrat, wiederholte sich dieser bei Geschwistern Ihres Vaters? | **ja**  **nein**  **unbekannt** | **ja**  **nein**  **unbekannt** | **ja**  **nein**  **unbekannt** | **ja**  **nein**  **unbekannt** | **ja**  **nein**  **unbekannt** |
| **D13** | Wenn ja, **wie oft** und **wann** trat insgesamt der Leistenbruch auf der linken Seite bei Geschwistern Ihres Vaters auf? |  |  |  |  |  |

**Nur wenn bei Geschwistern Ihres Vaters ein Narben- oder Leistenbruch wiederholt auftrat, ist es notwendig auch die folgenden Fragen zu beantworten. Ansonsten bitte weiter auf Seite 21!**

| **Nr.** | **Erkrankung** | **1.**  **Schwester/**  **Bruder Ihres Vaters** | **2.**  **Schwester/**  **Bruder Ihres Vaters** | **3.**  **Schwester/**  **Bruder Ihres Vaters** | **4.**  **Schwester/**  **Bruder Ihres Vaters** | **5.**  **Schwester/**  **Bruder Ihres Vaters** |
| --- | --- | --- | --- | --- | --- | --- |
| **D14** | Litten Geschwister Ihres Vaters über längere Zeit an einer **chronischen Verstopfung** (Obstipation) , die von einem Arzt festgestellt und medikamentös behandelt wurde? | **ja**  **nein**  **unbekannt** | **ja**  **nein**  **unbekannt** | **ja**  **nein**  **unbekannt** | **ja**  **nein**  **unbekannt** | **ja**  **nein**  **unbekannt** |
| **D15** | Wenn eine dauerhafte Verstopfung festgestellt wurde, **wie lange** dauerte die Verstopfung bei Geschwistern Ihres Vaters ungefähr an? |  |  |  |  |  |
| **D16** | Litten Geschwister Ihres Vaters über einen längeren Zeitraum an einer **chronischen Bronchitis**, die ärztlich nachgewiesen und durch eine Arzt medikamentös behandelt wurde? | **ja**  **nein**  **unbekannt** | **ja**  **nein**  **unbekannt** | **ja**  **nein**  **unbekannt** | **ja**  **nein**  **unbekannt** | **ja**  **nein**  **unbekannt** |
| **D17** | **Wie lange** dauerte die chronische  Bronchitis bei Geschwistern Ihres Vaters an? |  |  |  |  |  |
| **D18** | Wann stellte ein Arzt erstmals eine so **langwierige Bronchitis** bei Geschwistern Ihres Vaters fest? |  |  |  |  |  |
| **D19** | Stellte man bei Geschwistern Ihres Vaters eine **Zuckererkrankung** (Diabetes) fest? | **ja**  **nein**  **unbekannt** | **ja**  **nein**  **unbekannt** | **ja**  **nein**  **unbekannt** | **ja**  **nein**  **unbekannt** | **ja**  **nein**  **unbekannt** |
| **Nr.** | **Erkrankung** | **1.**  **Schwester/**  **Bruder Ihres Vaters** | **2.**  **Schwester/**  **Bruder Ihres Vaters** | **3.**  **Schwester/**  **Bruder Ihres Vaters** | **4.**  **Schwester/**  **Bruder Ihres Vaters** | **5.**  **Schwester/**  **Bruder Ihres Vaters** |
| **D20** | Wenn ja , sind Geschwister Ihres Vaters aufgrund dieser Zuckererkrankung **insulinpflichtig oder nicht**? | **ja**  **nein**  **unbekannt** | **ja**  **nein**  **unbekannt** | **ja**  **nein**  **unbekannt** | **ja**  **nein**  **unbekannt** | **ja**  **nein**  **unbekannt** |
| **D21** | Geben Sie bitte, wenn Sie können, das **Datum der Erstfeststellung der Diabetes** durch einen Arzt an! |  |  |  |  |  |
| **D22** | Stellte ein Arzt eine **Koronare Herzerkrankung**  bei Geschwistern Ihres Vaters fest? | **ja**  **nein**  **unbekannt** | **ja**  **nein**  **unbekannt** | **ja**  **nein**  **unbekannt** | **ja**  **nein**  **unbekannt** | **ja**  **nein**  **unbekannt** |
| **D23** | Geben Sie bitte, wenn Sie können, das **Datum der Erstfeststellung der koronaren Herzerkrankung** durch einen Arzt an! |  |  |  |  |  |
| **D24** | Leiden Geschwister Ihres Vaters an **Bluthochdruck** (Hypertonie), der medikamentös eingestellt ist? | **ja**  **nein**  **unbekannt** | **ja**  **nein**  **unbekannt** | **ja**  **nein**  **unbekannt** | **ja**  **nein**  **unbekannt** | **ja**  **nein**  **unbekannt** |
| **D25** | Geben Sie bitte, wenn Sie können, das **Datum der Erstfeststellung des Bluthochdrucks** durch einen Arzt an! |  |  |  |  |  |
| **D26** | Wurde bei Brüdern Ihres Vaters eine **Vergrößerung der Prostata** durch einen Arzt festgestellt ? | **ja**  **nein**  **unbekannt** | **ja**  **nein**  **unbekannt** | **ja**  **nein**  **unbekannt** | **ja**  **nein**  **unbekannt** | **ja**  **nein**  **unbekannt** |
| **D27** | Geben Sie bitte, wenn Sie können, das **Datum der Erstfeststellung der Prostatavergrößerung** durch einen Arzt an! |  |  |  |  |  |
| **D28** | Stellte man bei Geschwistern Ihres Vaters einen **bösartigen Tumor in der Bauchregion** fest? | **ja**  **nein**  **unbekannt** | **ja**  **nein**  **unbekannt** | **ja**  **nein**  **unbekannt** | **ja**  **nein**  **unbekannt** | **ja**  **nein**  **unbekannt** |
| **D29** | Wenn ja, kennen Sie den **Namen des Tumors**? Bitte geben Sie diesen an oder beschreiben Sie den Tumor, wenn Sie können, näher! **Wurde operiert?** |  |  |  |  |  |
| **D30** | Geben Sie bitte das **ungefähre Datum der ersten ärztlichen Feststellung des bösartigen Tumors** an ! |  |  |  |  |  |
| **Nr.** | **Erkrankung** | **1.**  **Schwester/**  **Bruder Ihres Vaters** | **2.**  **Schwester/**  **Bruder Ihres Vaters** | **3.**  **Schwester/**  **Bruder Ihres Vaters** | **4.**  **Schwester/**  **Bruder Ihres Vaters** | **5.**  **Schwester/**  **Bruder Ihres Vaters** |
| **D31** | Stellte ein Arzt bei Geschwistern Ihres Vaters **sonstige Erkrankungen** fest? | **ja**  **nein**  **unbekannt** | **ja**  **nein**  **unbekannt** | **ja**  **nein**  **unbekannt** | **ja**  **nein**  **unbekannt** | **ja**  **nein**  **unbekannt** |
| **D32** | Wenn ja, schreiben Sie bitte den Namen der Erkrankung und das entsprechende erstmalige Feststellungsdatum bei Geschwistern Ihres Vaters hier auf! | | | | | |

E

**Eigene Geschwister**

**Falls Sie keine Geschwister haben, setzen Sie bitte auf der Seite 25 mit den Angaben zu den eigenen Kindern fort.**

**Sicherlich sind Ihnen manche Daten Ihrer Geschwister nicht bekannt! Versuchen Sie bitte trotzdem die Angaben zu machen, die Ihnen zur Verfügung stehen! Ansonsten kreuzen Sie bitte „unbekannt“ an oder tragen Sie ein „?“ in das Kästchen ein!**

**E1) Wie viele Geschwister haben Sie (bitte auch Verstorbene angeben)?____________________**

| **Nr.** | **Frage** | **1.**  **Schwester/**  **Bruder** | **2.**  **Schwester/**  **Bruder** | **3.**  **Schwester/**  **Bruder** | **4.**  **Schwester/**  **Bruder** | **5.**  **Schwester/**  **Bruder** |
| --- | --- | --- | --- | --- | --- | --- |
| **E2** | **Geburtsdatum** Ihrer Geschwister? |  |  |  |  |  |
| **E3** | **Geschlecht** Ihrer Geschwister? | **männlich**  **weiblich** | **männlich**  **weiblich** | **männlich**  **weiblich** | **männlich**  **weiblich** | **männlich**  **weiblich** |
| **E4** | Wurde bei Ihren Geschwistern jemals eine **Bauchoperation** durchgeführt? [wenn nicht, weiter mit Frage E8] | **ja**  **nein**  **unbekannt** | **ja**  **nein**  **unbekannt** | **ja**  **nein**  **unbekannt** | **ja**  **nein**  **unbekannt** | **ja**  **nein**  **unbekannt** |
| **E5** | Wenn ja, um **welche** Operation handelte es sich? | **1.**  **2.**  **3.** | **1.**  **2.**  **3.** | **1.**  **2.**  **3.** | **1.**  **2.**  **3.** | **1.**  **2.**  **3.** |
| **E6** | Trat an den von Ihnen angeführten Operationsnarben bei Ihren Geschwistern schon einmal ein **Narbenbruch** auf? [wenn nicht weiter mit Frage E8] | **ja**  **nein**  **unbekannt** | **ja**  **nein**  **unbekannt** | **ja**  **nein**  **unbekannt** | **ja**  **nein**  **unbekannt** | **ja**  **nein**  **unbekannt** |
| **E7** | Wenn ja, **wie oft** und **wann** trat der Bruch an den jeweiligen Narben Ihrer Geschwister auf? | **1.Narbe:**  **2.Narbe:**  **3.Narbe:** | **1.Narbe:**  **2.Narbe:**  **3.Narbe:** | **1.Narbe:**  **2.Narbe:**  **3.Narbe:** | **1.Narbe:**  **2.Narbe:**  **3.Narbe:** | **1.Narbe:**  **2.Narbe:**  **3.Narbe:** |
| **E8** | Trat bei Ihren Geschwistern schon einmal ein **Leistenbruch** auf?  [wenn nicht weiter mit Frage E14] | **ja**  **nein**  **unbekannt** | **ja**  **nein**  **unbekannt** | **ja**  **nein**  **unbekannt** | **ja**  **nein**  **unbekannt** | **ja**  **nein**  **unbekannt** |
| **E9** | Wenn ja, auf welcher **Seite**?  [wenn nur links, weiter mit Frage E12, wenn nur rechts oder beidseitig weiter mit Frage E10] | ** links**  ** rechts**  ** beide Seiten** | ** links**  ** rechts**  ** beide Seiten** | ** links**  ** rechts**  ** beide Seiten** | ** links**  ** rechts**  ** beide Seiten** | ** links**  ** rechts**  ** beide Seiten** |
| **E10** | Wenn der **Leistenbruch rechts** auftrat, wiederholte sich dieser bei Ihren Geschwistern? | **ja**  **nein**  **unbekannt** | **ja**  **nein**  **unbekannt** | **ja**  **nein**  **unbekannt** | **ja**  **nein**  **unbekannt** | **ja**  **nein**  **unbekannt** |

| **Nr.** | **Frage** | **1.**  **Schwester/**  **Bruder** | **2.**  **Schwester/**  **Bruder** | **3.**  **Schwester/**  **Bruder** | **4.**  **Schwester/**  **Bruder** | **5.**  **Schwester/**  **Bruder** |
| --- | --- | --- | --- | --- | --- | --- |
| **E11** | Wenn ja, **wie oft** und **wann** trat der Leistenbruch insgesamt auf der rechten Seite bei Ihren Geschwistern auf? |  |  |  |  |  |
| **E12** | Wenn der **Leistenbruch links** auftrat, wiederholte sich dieser bei Ihren Geschwistern? | **ja**  **nein**  **unbekannt** | **ja**  **nein**  **unbekannt** | **ja**  **nein**  **unbekannt** | **ja**  **nein**  **unbekannt** | **ja**  **nein**  **unbekannt** |
| **E13** | Wenn ja**, wie oft** und **wann** trat insgesamt der Leistenbruch auf der linken Seite bei Ihren Geschwistern auf? |  |  |  |  |  |

**Nur wenn bei Geschwistern Ihres Vaters ein Narben- oder Leistenbruch wiederholt auftrat, ist es notwendig auch die folgenden Fragen zu beantworten. Ansonsten bitte weiter auf Seite 25 !**

| **Nr.** | **Erkrankung** | **1.**  **Schwester/**  **Bruder** | **2.**  **Schwester/**  **Bruder** | **3.**  **Schwester/**  **Bruder** | **4.**  **Schwester/**  **Bruder** | **5.**  **Schwester/**  **Bruder** |
| --- | --- | --- | --- | --- | --- | --- |
| **E14** | Litten Ihre Geschwister über längere Zeit an einer **chronischen Verstopfung** (Obstipation) , die von einem Arzt festgestellt und medikamentös behandelt wurde? | **ja**  **nein**  **unbekannt** | **ja**  **nein**  **unbekannt** | **ja**  **nein**  **unbekannt** | **ja**  **nein**  **unbekannt** | **ja**  **nein**  **unbekannt** |
| **E15** | Wenn eine dauerhafte Verstopfung festgestellt wurde, **wie lange** dauerte die Verstopfung bei Ihren Geschwistern ungefähr an? |  |  |  |  |  |
| **E16** | Litten Ihre Geschwister über einen längeren Zeitraum an einer **chronischen Bronchitis**, die ärztlich nachgewiesen und durch eine Arzt medikamentös behandelt wurde? | **ja**  **nein**  **unbekannt** | **ja**  **nein**  **unbekannt** | **ja**  **nein**  **unbekannt** | **ja**  **nein**  **unbekannt** | **ja**  **nein**  **unbekannt** |
| **E17** | **Wie lange** dauerte die chronische  Bronchitis bei Ihren Geschwistern an? |  |  |  |  |  |
| **E18** | **Wann** stellte ein Arzt erstmals eine so langwierige Bronchitis bei Ihren Geschwistern fest? |  |  |  |  |  |
| **E19** | Stellte man bei Ihren Geschwistern eine **Zuckererkrankung** (Diabetes) fest? | **ja**  **nein**  **unbekannt** | **ja**  **nein**  **unbekannt** | **ja**  **nein**  **unbekannt** | **ja**  **nein**  **unbekannt** | **ja**  **nein**  **unbekannt** |
| **E20** | Wenn ja , sind Ihre Geschwister aufgrund dieser Zuckererkrankung **insulinpflichtig oder nicht**? | **ja**  **nein**  **unbekannt** | **ja**  **nein**  **unbekannt** | **ja**  **nein**  **unbekannt** | **ja**  **nein**  **unbekannt** | **ja**  **nein**  **unbekannt** |
| **Nr.** | **Erkrankung** | **1.**  **Schwester/**  **Bruder** | **2.**  **Schwester/**  **Bruder** | **3.**  **Schwester/**  **Bruder** | **4.**  **Schwester/**  **Bruder** | **5.**  **Schwester/**  **Bruder** |
| **E21** | Geben Sie bitte, wenn Sie können, das **Datum der Erstfeststellung der Diabetes** durch einen Arzt an! |  |  |  |  |  |
| **E22** | Stellte ein Arzt eine **Koronare Herzerkrankung**  bei Ihren Geschwistern fest? | **ja**  **nein**  **unbekannt** | **ja**  **nein**  **unbekannt** | **ja**  **nein**  **unbekannt** | **ja**  **nein**  **unbekannt** | **ja**  **nein**  **unbekannt** |
| **E23** | Geben Sie bitte, wenn Sie können, das **Datum der Erstfeststellung der koronaren Herzerkrankung** durch einen Arzt an! |  |  |  |  |  |
| **E24** | Leiden Ihre Geschwister an **Bluthochdruck** (Hypertonie), der medikamentös eingestellt ist? | **ja**  **nein**  **unbekannt** | **ja**  **nein**  **unbekannt** | **ja**  **nein**  **unbekannt** | **ja**  **nein**  **unbekannt** | **ja**  **nein**  **unbekannt** |
| **E25** | Geben Sie bitte, wenn Sie können, das **Datum der Erstfeststellung des Bluthochdrucks** durch einen Arzt an! |  |  |  |  |  |
| **E26** | Wurde bei Ihren Brüdern eine **Vergrößerung der Prostata** durch einen Arzt festgestellt ? | **ja**  **nein**  **unbekannt** | **ja**  **nein**  **unbekannt** | **ja**  **nein**  **unbekannt** | **ja**  **nein**  **unbekannt** | **ja**  **nein**  **unbekannt** |
| **E27** | Geben Sie bitte, wenn Sie können, das Datum der Erstfeststellung der Prostatavergrößerung durch einen Arzt an! |  |  |  |  |  |
| **E28** | Stellte man bei Ihren Geschwistern einen **bösartigen Tumor in der Bauchregion** fest? | **ja**  **nein**  **unbekannt** | **ja**  **nein**  **unbekannt** | **ja**  **nein**  **unbekannt** | **ja**  **nein**  **unbekannt** | **ja**  **nein**  **unbekannt** |
| **E29** | Wenn ja, kennen Sie den **Namen des Tumors**? Bitte geben Sie diesen an oder beschreiben Sie den Tumor, wenn Sie können, näher! **Wurde operiert?** |  |  |  |  |  |
| **E30** | Geben Sie bitte das **ungefähre Datum der ersten ärztlichen Feststellung des bösartigen Tumors** an ! |  |  |  |  |  |
| **E31** | Stellte ein Arzt bei Ihren Geschwistern sonstige Erkrankungen fest? | **ja**  **nein**  **unbekannt** | **ja**  **nein**  **unbekannt** | **ja**  **nein**  **unbekannt** | **ja**  **nein**  **unbekannt** | **ja**  **nein**  **unbekannt** |
| **E32** | Wenn ja, schreiben Sie bitte den **Namen der Erkrankung** und das entsprechende **erstmalige Feststellungsdatum** bei Ihren Geschwistern umseitig auf! | | | | | |

F

**Eigene Kinder**

**Falls Sie keine Kinder haben, haben Sie die Beantwortung des Fragebogens beendet. Vielen Dank für Ihre engagierte Mitarbeit !**

**F1) Wie viele Kinder haben Sie (bitte auch Verstorbene angeben)?_________________________**

| **Nr.** | **Frage** | **1.Kind** | **2.Kind** | **3.Kind** | **4.Kind** | **5.Kind** |
| --- | --- | --- | --- | --- | --- | --- |
| **F2** | **Geburtsdatum** Ihrer Kinder? |  |  |  |  |  |
| **F3** | **Geschlecht** Ihrer Kinder? | **männlich**  **weiblich** | **männlich**  **weiblich** | **männlich**  **weiblich** | **männlich**  **weiblich** | **männlich**  **weiblich** |
| **F4** | Wurde bei Ihren Kindern jemals eine **Bauchoperation** durchgeführt? [wenn nicht, weiter mit Frage F8] | **ja**  **nein**  **unbekannt** | **ja**  **nein**  **unbekannt** | **ja**  **nein**  **unbekannt** | **ja**  **nein**  **unbekannt** | **ja**  **nein**  **unbekannt** |
| **F5** | Wenn ja, um **welche** Operation handelte es sich? | **1.**  **2.**  **3.** | **1.**  **2.**  **3.** | **1.**  **2.**  **3.** | **1.**  **2.**  **3.** | **1.**  **2.**  **3.** |
| **F6** | Trat an den von Ihnen angeführten Operationsnarben bei Ihren Kindern schon einmal ein **Narbenbruch** auf? [wenn nicht weiter mit Frage F8] | **ja**  **nein**  **unbekannt** | **ja**  **nein**  **unbekannt** | **ja**  **nein**  **unbekannt** | **ja**  **nein**  **unbekannt** | **ja**  **nein**  **unbekannt** |
| **F7** | Wenn ja, **wie oft** und **wann** trat der Bruch an den jeweiligen Narben Ihrer Kinder auf? | **1.Narbe:**  **2.Narbe:**  **3.Narbe:** | **1.Narbe:**  **2.Narbe:**  **3.Narbe:** | **1.Narbe:**  **2.Narbe:**  **3.Narbe:** | **1.Narbe:**  **2.Narbe:**  **3.Narbe:** | **1.Narbe:**  **2.Narbe:**  **3.Narbe:** |
| **F8** | Trat bei Ihren Kindern schon einmal ein **Leistenbruch** auf?  [wenn nicht weiter mit Frage F14] | **ja**  **nein**  **unbekannt** | **ja**  **nein**  **unbekannt** | **ja**  **nein**  **unbekannt** | **ja**  **nein**  **unbekannt** | **ja**  **nein**  **unbekannt** |
| **F9** | Wenn ja, auf welcher **Seite**?  [wenn nur links, weiter mit Frage F12, wenn nur rechts oder beidseitig weiter mit Frage F10] | ** links**  ** rechts**  ** beide Seiten** | ** links**  ** rechts**  ** beide Seiten** | ** links**  ** rechts**  ** beide Seiten** | ** links**  ** rechts**  ** beide Seiten** | ** links**  ** rechts**  ** beide Seiten** |
| **F10** | Wenn der **Leistenbruch rechts** auftrat, wiederholte sich dieser bei Ihren Kindern? | **ja**  **nein**  **unbekannt** | **ja**  **nein**  **unbekannt** | **ja**  **nein**  **unbekannt** | **ja**  **nein**  **unbekannt** | **ja**  **nein**  **unbekannt** |

| **Nr.** | **Frage** | **1.Kind** | **2.Kind** | **3.Kind** | **4.Kind** | **5.Kind** |
| --- | --- | --- | --- | --- | --- | --- |
| **F11** | Wenn ja, **wie oft** und **wann** trat der Leistenbruch insgesamt auf der rechten Seite bei Ihren Kindern auf ? |  |  |  |  |  |
| **F12** | Wenn der **Leistenbruch links** auftrat, wiederholte sich dieser bei Ihren Kindern? | **ja**  **nein**  **unbekannt** | **ja**  **nein**  **unbekannt** | **ja**  **nein**  **unbekannt** | **ja**  **nein**  **unbekannt** | **ja**  **nein**  **unbekannt** |
| **F13** | Wenn ja, **wie oft** und **wann** trat insgesamt der Leistenbruch auf der linken Seite bei Ihren Kindern auf? |  |  |  |  |  |

**Nur wenn bei Ihren Kindern ein Narben- oder Leistenbruch wiederholt auftrat, ist es notwendig auch die folgenden Fragen zu beantworten. Ansonsten haben Sie nun alle Fragen beantwortet! Vielen Dank!**

| **Nr.** | **Erkrankung** | **1.Kind** | **2.Kind** | **3.Kind** | **4.Kind** | **5.Kind** |
| --- | --- | --- | --- | --- | --- | --- |
| **F14** | Litten Ihre Kinder über längere Zeit an einer **chronischen Verstopfung** (Obstipation) , die von einem Arzt festgestellt und medikamentös behandelt wurde? | **ja**  **nein**  **unbekannt** | **ja**  **nein**  **unbekannt** | **ja**  **nein**  **unbekannt** | **ja**  **nein**  **unbekannt** | **ja**  **nein**  **unbekannt** |
| **F15** | Wenn eine dauerhafte Verstopfung festgestellt wurde**, wie lange** dauerte die Verstopfung bei Ihren Kindern ungefähr an? |  |  |  |  |  |
| **F16** | Litten Ihre Kinder über einen längeren Zeitraum an einer **chronischen Bronchitis,** die ärztlich nachgewiesen und durch eine Arzt medikamentös behandelt wurde? | **ja**  **nein**  **unbekannt** | **ja**  **nein**  **unbekannt** | **ja**  **nein**  **unbekannt** | **ja**  **nein**  **unbekannt** | **ja**  **nein**  **unbekannt** |
| **F17** | **Wie lange** dauerte die chronische  Bronchitis bei Ihren Kindern an? |  |  |  |  |  |
| **F18** | **Wann** stellte ein Arzt erstmals eine so langwierige Bronchitis bei Ihren Kindern fest? |  |  |  |  |  |
| **F19** | Stellte man bei Ihren Kindern eine **Zuckererkrankung** (Diabetes) fest? | **ja**  **nein**  **unbekannt** | **ja**  **nein**  **unbekannt** | **ja**  **nein**  **unbekannt** | **ja**  **nein**  **unbekannt** | **ja**  **nein**  **unbekannt** |
| **F20** | Wenn ja , sind Ihre Kinder aufgrund dieser Zuckererkrankung **insulinpflichtig oder nicht**? | **ja**  **nein**  **unbekannt** | **ja**  **nein**  **unbekannt** | **ja**  **nein**  **unbekannt** | **ja**  **nein**  **unbekannt** | **ja**  **nein**  **unbekannt** |
| **F21** | Geben Sie bitte, wenn Sie können, das **Datum der Erstfeststellung der Diabetes** durch einen Arzt an! |  |  |  |  |  |
| **Nr.** | **Erkrankung** | **1.Kind** | **2.Kind** | **3.Kind** | **4.Kind** | **5.Kind** |
| **F22** | Stellte ein Arzt eine **Koronare Herzerkrankung** bei Ihren Kindern fest? | **ja**  **nein**  **unbekannt** | **ja**  **nein**  **unbekannt** | **ja**  **nein**  **unbekannt** | **ja**  **nein**  **unbekannt** | **ja**  **nein**  **unbekannt** |
| **F23** | Geben Sie bitte, wenn Sie können, das **Datum der Erstfeststellung der koronaren Herzerkrankung** durch einen Arzt an! |  |  |  |  |  |
| **F24** | Leiden Ihre Kinder an **Bluthochdruck** (Hypertonie), der medikamentös eingestellt ist? | **ja**  **nein**  **unbekannt** | **ja**  **nein**  **unbekannt** | **ja**  **nein**  **unbekannt** | **ja**  **nein**  **unbekannt** | **ja**  **nein**  **unbekannt** |
| **F25** | Geben Sie bitte, wenn Sie können, das **Datum der Erstfeststellung des Bluthochdrucks** durch einen Arzt an! |  |  |  |  |  |
| **F26** | Wurde bei Ihren Söhnen eine **Vergrößerung der Prostata** durch einen Arzt festgestellt ? | **ja**  **nein**  **unbekannt** | **ja**  **nein**  **unbekannt** | **ja**  **nein**  **unbekannt** | **ja**  **nein**  **unbekannt** | **ja**  **nein**  **unbekannt** |
| **F27** | Geben Sie bitte, wenn Sie können, das **Datum der Erstfeststellung der Prostatavergrößerung** durch einen Arzt an! |  |  |  |  |  |
| **F28** | Stellte man bei Ihren Kindern einen **bösartigen Tumor in der Bauchregion** fest? | **ja**  **nein**  **unbekannt** | **ja**  **nein**  **unbekannt** | **ja**  **nein**  **unbekannt** | **ja**  **nein**  **unbekannt** | **ja**  **nein**  **unbekannt** |
| **F29** | Wenn ja, kennen Sie den **Namen des Tumors**? Bitte geben Sie diesen an oder beschreiben Sie den Tumor, wenn Sie können, näher! **Wurde operiert**? |  |  |  |  |  |
| **F30** | Geben Sie bitte das ungefähre **Datum der ersten ärztlichen Feststellung des bösartigen Tumors** an ! |  |  |  |  |  |
| **F31** | Stellte ein Arzt bei Ihren Kindern **sonstige Erkrankungen** fest? | **ja**  **nein**  **unbekannt** | **ja**  **nein**  **unbekannt** | **ja**  **nein**  **unbekannt** | **ja**  **nein**  **unbekannt** | **ja**  **nein**  **unbekannt** |
| **F32** | Wenn ja, schreiben Sie bitte den **Namen der Erkrankung** und das entsprechende **erstmalige Feststellungsdatum** bei Ihren Kindern umseitig auf! | | | | | |

*Wir danken Ihnen für Ihre engagierte Mitarbeit !*
